# Antibiotic-Resistant Vibrio cholerae O1 and Its SXT Elements Associated with Two Cholera Epidemics in Kenya in 2007 to 2010 and 2015 to 2016

**DOI:** 10.1128/spectrum.04140-22

**Published:** 2023-05-01

**Authors:** Mohammad Monir Shah, Martin Bundi, Cyrus Kathiiko, Sora Guyo, Amina Galata, Gabriel Miringu, Yoshio Ichinose, Lay-Myint Yoshida

**Affiliations:** a Department of Pediatric Infectious Diseases, Institute of Tropical Medicine, Nagasaki University, Nagasaki, Japan; b Nagasaki University Institute of Tropical Medicine–Kenya Medical Research Institute Project, Nairobi, Kenya; c Kenya Medical Research Institute, Nairobi, Kenya; University of Washington

**Keywords:** *V. cholerae* O1, cholera epidemic, multidrug-resistant strain, antibiotic-resistant mechanisms

## Abstract

Multidrug-resistant Vibrio cholerae O1 strains have long been observed in Africa, and strains exhibiting new resistance phenotypes have emerged during recent epidemics in Kenya. This study aimed to determine the epidemiological aspects, drug resistance patterns, and genetic elements of V. cholerae O1 strains isolated from two cholera epidemics in Kenya between 2007 and 2010 and between 2015 and 2016. A total of 228 V. cholerae O1 strains, including 226 clinical strains isolated from 13 counties in Kenya during the 2007–2010 and 2015–2016 cholera epidemics and two environmental isolates (from shallow well water and spring water isolates) isolated from Pokot and Kwale Counties, respectively, in 2010 were subjected to biotyping, serotyping, and antimicrobial susceptibility testing, including the detection of antibiotic resistance genes and mobile genetic elements. All V. cholerae isolates were identified as El Tor biotypes and susceptible to ceftriaxone, gentamicin, and ciprofloxacin. The majority of isolates were resistant to trimethoprim-sulfamethoxazole (94.6%), streptomycin (92.8%), and nalidixic acid (64.5%), while lower resistance was observed against ampicillin (3.6%), amoxicillin (4.2%), chloramphenicol (3.0%), and doxycycline (1.8%). Concurrently, the integrating conjugative (SXT) element was found in 95.5% of the V. cholerae isolates; conversely, class 1, 2, and 3 integrons were absent. Additionally, 64.5% of the isolates exhibited multidrug resistance patterns. Antibiotic-resistant gene clusters suggest that environmental bacteria may act as cassette reservoirs that favor resistant pathogens. On the other hand, the 2015–2016 epidemic strains were found susceptible to most antibiotics except nalidixic acid. This revealed the replacement of multidrug-resistant strains exhibiting new resistance phenotypes that emerged after Kenya's 2007–2010 epidemic.

**IMPORTANCE** Kenya is a country where cholera is endemic; it has experienced three substantial epidemics over the past few decades, but there are limited data on the drug resistance patterns of V. cholerae at the national level. To the best of our knowledge, this is the first study to investigate the antimicrobial susceptibility profiles of V. cholerae O1 strains isolated from two consecutive epidemics and to examine their associated antimicrobial genetic determinants. Our study results revealed two distinct antibiotic resistance trends in two separate epidemics, particularly trends for multidrug-associated mobile genetic elements and chromosomal mutation-oriented resistant strains from the 2007–2010 epidemic. In contrast, only nalidixic acid-associated chromosomal mutated strains were isolated from the 2015–2016 epidemic. This study also found similar patterns of antibiotic resistance in environmental and clinical strains. Continuous monitoring is needed to control emerging multidrug-resistant isolates in the future.

## INTRODUCTION

Cholera is a life-threatening acute watery diarrheal disease caused by ingesting food or water contaminated with the bacterium Vibrio cholerae O1 or O139 and is responsible for long-term public health crises in many developing countries, particularly in South America, Southeast Asia, and Africa. Annually, cholera causes approximately 1.3 to 4.0 million cases and 21,000 to 143,000 deaths worldwide (1). Since 1971, when the seventh pandemic of V. cholerae O1 El Tor biotype reached Kenya, the pandemic has persisted and caused multiple outbreaks (2). According to the World Health Organization (WHO), cholera cases have been reported in Kenya from 1974 to 1989, with an average case fatality rate of 3.6% (3). Furthermore, between 1997 and 2016, >79,000 clinically confirmed cholera cases and >2,800 deaths were reported in Kenya (2). Also, during the 2008 cholera epidemic in Zimbabwe, >90,000 suspected cholera cases and >4,000 deaths were reported (4). Moreover, a recent study reported that the cholera outbreak incidence has increased in sub-Saharan African countries (5).

Cholera spread widely across Africa during the seventh pandemic, but little is known about the origin of this continental spread. Epidemiological data from individual countries have provided insight into cholera transmission (6). Conventional methods, such as O-antigen-based serotyping (Ogawa and Inaba) theoretically provided additional data for lineage differentiation across outbreak regions of Africa; however, this method proved unreliable, and different molecular approaches were used to examine the propagation routes (7, 8). Recent genomic analysis has identified the two El Tor strains associated with the invasion of Africa in 1970: one in West Africa (serotype Ogawa) and the other in East Africa (serotype Inaba) (9). The Ogawa and Inaba serotypes differ by the presence of a methyl group on the terminal sugar of the Ogawa O-antigen that is absent in Inaba strains. This methylation is catalyzed by a methyltransferase encoded by the *wbeT* gene (previously called *rfbT*), which is disrupted by a mutation in Inaba strains (10). In addition, a third serotype called Hikojima is rare and known as an unstable transitional serotype observed when the strain undergoes a serotype switch from Ogawa to Inaba (10). Seroconversion is a natural process observed in regions where cholera is endemic, possibly through effects on the host's immune response (10).

Rehydration therapy is the primary treatment for cholera, though antibiotics are recommended to reduce the severity and duration of diarrhea (11). The emergence of antibiotic-resistant pathogens has become a serious global health issue. This resistance crisis has been exacerbated by the intensive use of antibiotics in the health and agriculture sectors and the emission of antibiotic-related industrial wastes into the environment (12). Antimicrobial-resistant V. cholerae has rapidly spread worldwide, particularly in Africa (5, 9), and multidrug-resistant (MDR) strains have emerged in recent years (9). Since the 1960s, tetracycline, chloramphenicol, and trimethoprim-sulfamethoxazole have been used effectively to treat cholera (13). Also, β-lactam antibiotics are widely prescribed for many bacterial infections. Therefore, excessive use of these antibiotics to treat infectious diseases other than cholera may lead to this resistance.

Additionally, recent studies have reported that V. cholerae can acquire antibiotic resistance through chromosomal mutation or acquisition of resistance genes via mobile genetic elements, such as conjugative plasmids, integrons, transposons, or integrating conjugative elements (ICEs) (14–16). Integrons are genetic elements often found within conjugative plasmids, allowing bacteria to capture mobile gene cassettes via site-specific recombination. Most of these gene cassettes encode antibiotic resistance genes (14). ICEs and plasmids are transmitted via conjugation; however, unlike plasmids, ICEs integrate and replicate along with the chromosome and contain regulatory genes to control their excision from the chromosome and conjugative transfer (17). ICEs can carry multiple drug resistance genes and are responsible for spreading antimicrobial drug resistance in various Gram-positive and Gram-negative bacteria (17). The SXT element is an ~100-kb ICE that has played a significant role in spreading antimicrobial resistance in the seventh-pandemic V. cholerae. SXT encodes resistance to multiple antibiotics, including streptomycin, sulfamethoxazole, and trimethoprim (termed SXT), which have previously been useful in treating cholera (17). SXT was first identified in a V. cholerae O139 clinical strain (MO10) isolated in India (18); this strain was later used as a reference for comparison with other SXT-like elements (17). Subsequently, several SXT elements have been reported in many clinical and environmental strains of isolated V. cholerae O1 globally (15). Besides V. cholerae, the SXT element (ICEP*al*Ban1) has also been isolated from clinical strains of Providencia alcalifaciens in Bangladesh. The SXT element (ICE*Pal*Ban1) is closely related to the element ICE*Vch*Ind1 (V. cholerae O1) (15), indicating its host range is not only limited to *Vibrio* species. It has also been detected in Photobacterium damselae and Shewanella putrefaciens (17).

Kenya has experienced sporadic cholera cases almost every year, including several waves of cholera epidemics. There is limited information on the drug resistance patterns of V. cholerae at the national level. We therefore sought to investigate the antimicrobial susceptibility patterns for 10 different antibiotics among V. cholerae O1 isolates collected from different regions of Kenya during the two epidemics from 2007 to 2010 and 2015 to 2016. Furthermore, this study investigated the genetic determinants associated with antibiotic resistance to understand the mechanism of the emergence of drug-resistant cholera strains.

## RESULTS

### Characteristics of the cholera epidemic cases.

**FIG 1:**
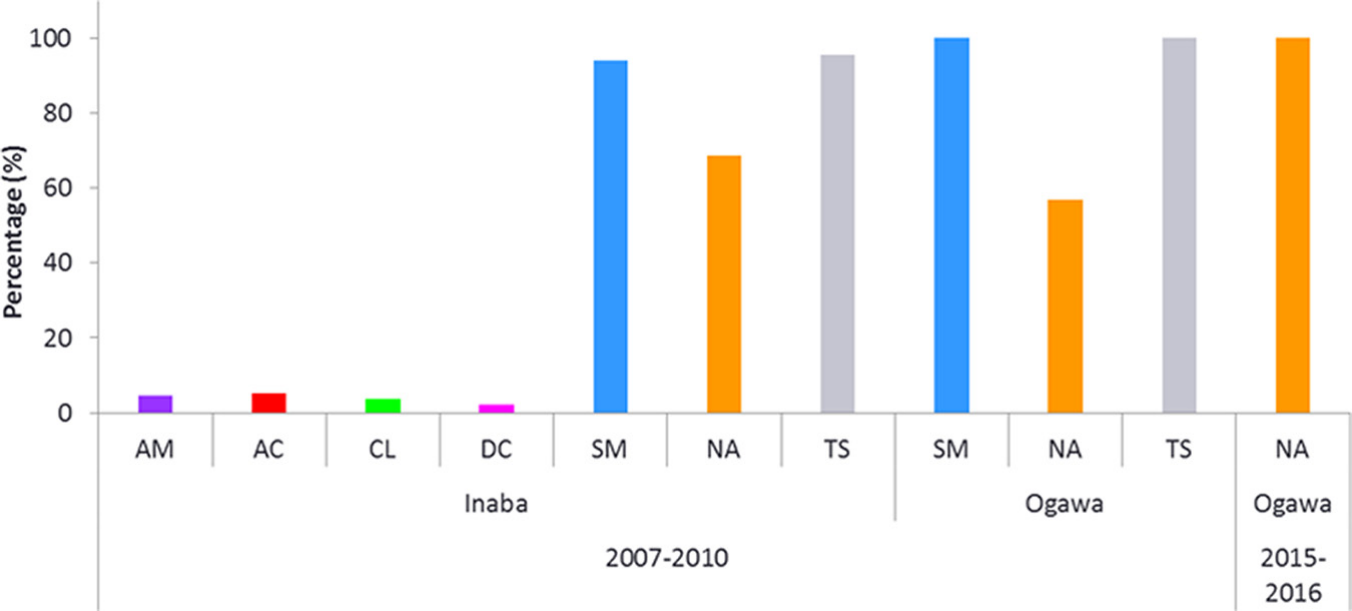
Antibiotic resistance patterns between Inaba and Ogawa serotype strains during the 2007–2010 and 2015–2016 cholera epidemics. The reported percentage for each type of antibiotic resistance was calculated based on the total number of each serotype (Inaba, *n* = 134; Ogawa, *n* = 30). AM, ampicillin; AC, amoxicillin; CL, chloramphenicol; DC, doxycycline; SM, streptomycin; NA, nalidixic acid; TS, trimethoprim-sulfamethoxazole.

A total of 522 samples were received from 13 counties in Kenya, of which 358 isolates were obtained during the 2007 to 2010 period and 164 isolates during 2015 to 2016, where 228 (43.7%) isolates were confirmed as V. cholerae O1 El Tor biotype (data not shown). Among these, 136 (81.9%) strains, including one environmental strain, were found to be serotype Inaba during the 2007–2010 epidemic, while 30 (18.1%) strains, including an environmental strain, were identified as serotype Ogawa. In addition, during the 2015–2016 epidemic, 62 (100%) strains were identified only as serotype Ogawa (Fig. 1; see also Table S1 in the supplemental material). In both epidemics, male patients (38.6% [2007–2010] and 59.7% [2015–2016]) were more likely to have diarrheal illness than female patients (28.9% [2007–2010] and 40.3% [2015–2016]) (Table 1). During the 2007–2010 epidemic, 54 (32.5%) isolates with gender information and 65 (39.2%) isolates with age information were omitted from data analysis due to missing data. Among the clinical isolates, different age groups of patients were observed and were characterized into five age groups ranging from ≤5 years to ≥60 years (Table 1). All age groups were infected with cholera; over 11% of children aged ≤5 years were found in both study periods. A relatively high percentage of cholera cases was observed in patients aged 21 to 40 years, and the incidence rate of infection gradually declined with age.

**TABLE 1:** Epidemiological characteristics of cases from two cholera epidemics in Kenya between 2007 and 2010 and between 2015 and 2016

Characteristic	No. (%) with characteristic from epidemic in:		*P* value
	2007–2010 (*N* = 166)	2015–2016 (*N* = 62)	
Gender			
Male	64 (38.6%)	37 (59.7%)	0.870
Female	48 (28.9%)	25 (40.3%)	
Other	54 (32.5%)		
Age (yrs)			
≤5	19 (11.4%)	7 (11.3%)	0.030
6–20	37 (22.3%)	13 (21%)	
21–40	28 (16.9%)	31 (50%)	
41–60	13 (7.8%)	10 (16.1%)	
≥60	4 (2.4%)	1 (1.6%)	
Other	65 (39.2%)		
Location			
Bungoma	17 (10.2%)		
Homa Bay	71 (42.8%)	11 (17.7%)	0.001
Isiolo	20 (12%)		
Kiambu	3 (1.8%)		
Kisumu	2 (1.2%)		
Kwale	16 (9.6%)		
Marsabit	3 (1.8%)		
Nakuru	5 (3%)		
Nairobi	12 (7.2%)	30 (48.4%)	<0.001
Siaya		3 (4.8%)	
Turkana	4 (2.4%)		
Wajir		18 (29%)	
Pokot	13 (7.8%)		

### Antibiotic resistance patterns.

Among 2007–2010 cholera epidemic isolates, two V. cholerae strains were sensitive to all antibiotics. During the 2007–2010 epidemic period, most of the isolates were resistant to trimethoprim-sulfamethoxazole (94.6%, 157/166), streptomycin (92.8%, 154/166), and nalidixic acid (64.5%, 107/166), while lower resistance was observed against ampicillin (3.6%, 6/166), amoxicillin (4.2%, 7/166), chloramphenicol (3.0%, 5/166), and doxycycline (1.8%, 3/166). Intermediate resistance to chloramphenicol was considerably higher than expected (42.2%, 70/166). In addition, 64.5% of V. cholerae strains, including two environmental isolates, were multidrug resistant (Table S1). On the other hand, all V. cholerae strains were susceptible to ceftriaxone, gentamicin, and ciprofloxacin (Table 2). Of the 62 strains in the 2015–2016 epidemic, 100% (62/62) were resistant to only nalidixic acid.

**TABLE 2:** Antimicrobial susceptibility of the 228 V. cholerae O1 strains during the 2007–2010 and 2015–2016 epidemics in Kenya

Epidemic	Antimicrobial agent(s)	No. (%) of isolates with indicated susceptibility^*a*^			MIC (μg/mL) data		
		S	I	R	MIC_50_	MIC_90_	MIC range
2007–2010	Ampicillin	157 (94.6)	3 (1.8)	6 (3.6)	1.84	3.95	0.25–256
	Amoxicillin	156 (95.6)	3 (1.8)	7 (4.2)	3.04	5.04	0.19–256
	Ceftriaxone	166 (100)	0	0	0.014	0.019	0.008–2
	Chloramphenicol	91 (54.8)	70 (42.2)	5 (3.0)	6.4	13.9	0.25–96
	Doxycycline	161 (97.0)	2 (1.2)	3 (1.8)	0.44	1.85	0.064–32
	Gentamycin	166 (100)	0	0	0.46	0.84	0.006–8
	Streptomycin	5 (3.0)	7 (4.2)	154 (92.8)	56.7	141.7	0.032–256
	Ciprofloxacin	166 (100)	0	0	0.02	0.42	0.002–1
	Nalidixic acid	52 (31.3)	7 (4.2)	107 (64.5)	≥256	≥256	0.125–256
	Trimethoprim-sulfamethoxazole	8 (4.8)	0	158 (95.2)	≥32	≥32	0.023–32
2015–2016	Nalidixic acid	0	0	62 (100)	≥256	≥256	0.125–256

a S, susceptible; I, intermediate; R, resistant.

### Distribution of antimicrobial resistance-conferring genes.

PCR test results showed that 95.5% of the 2007–2010 cholera epidemic isolates contained the SXT element integrase (int), among which genes for resistance to trimethoprim (*dfrA1*; 83.1%), sulfamethoxazole (*sul2*; 77.1%), streptomycin (*strA* [91.6%] and *strB* [80.7%]), and chloramphenicol (*floR*; 91.6%) were present, while none of the 2015–2016 epidemic isolates harbored a resistance genes other than the *dfrA1* gene (75.8%). PCR analysis of class 1, 2, and 3 integrons showed no amplification. Also, all strains were negative for the tetracycline resistance gene [*tet*(A)].

### Evaluation of quinolone susceptibility patterns.

Of the 226 strains, several representative strains were selected for amplification and sequencing of the quinolone resistance-determining region (QRDR) of DNA gyrase and topoisomerase genes, such as *gyrA*, *gyrB*, *parC*, and *parE*. Sequences of these genes have been deposited in the GenBank database (see “Data availability”). The sequence results showed that these isolates carried mutations in *gyrA* (with a Ser-to-Ile change at position 83 [Ser83Ile]) and *parC* (Ser85Leu) genes. No mutations were detected in either the *gyrB* or *parE* genes. All strains were PCR negative for the *qnrVC* gene.

## DISCUSSION

Cholera outbreaks have increased over the past few decades in sub-Saharan African countries such as Kenya, Tanzania, Rwanda, Burundi, Uganda, and the Democratic Republic of the Congo, possibly due to poor socio-economic conditions, inadequate sanitation, and poor access to safe drinking water or to El Niño warming events (19, 20). According to the WHO report, Kenya has experienced cholera outbreaks almost every year since 1971, including three substantial epidemics (during 1997 to 1999, 2007 to 2010, and 2015 to 2016) (3, 21). The emergence of multidrug-resistant V. cholerae is becoming another major health issue for Africa (9). This study investigated the antibiotic susceptibility patterns and genetic determinants of V. cholerae strains isolated during the two major epidemics in Kenya from 2007 to 2010 and 2015 to 16. All the strains isolated from the epidemics were V. cholerae serogroup O1 El Tor biotype. Serotype Inaba was observed initially (in 2007), but there was a shift from Inaba to Ogawa in late 2009 that persisted until the 2015–2016 epidemic; this has been reported in previous studies (19, 21). This fluctuation in serotype replacement was observed during prolonged episodes in areas where cholera is endemic (22). The reason for this fluctuation was possibly due to effects on the host's immune response (10). In both epidemics, the incidence of cholera was higher among men (57.1 to 59.7%) than women (40.3 to 42.9%); this is consistent with the findings of Mutonga et al. (19). We found that more than 11% of cases were among children age ≤5 years (Table 1). According to Dean et al. (23), three different incidence rates occurred among children aged 2 to 5 years in three different countries, with the highest incidence observed in the African site. This finding revealed the growing impression of the enormous cholera burden in Africa. However, more detailed studies involving data from a sufficiently long period are needed to understand the incidence of cholera in specific age groups in various communities over time.

Antibiotics have been used in combination with rehydration therapy for decades in the treatment of cholera. This approach shortens the duration of diarrhea and reduces the transmission of bacterial spread (11). However, the wide usage of antibiotics has increased antibiotic resistance in bacteria, leading to the emergence of multidrug-resistant (MDR) strains, which have become one of the most serious public health concerns worldwide. In this regard, areas where cholera is endemic, including African countries, are experiencing a rapid increase in the prevalence of antimicrobial-resistant cholera strains (9). Kitaoka and colleagues reported that V. cholerae becomes drug resistant by using efflux pumps, chromosomal mutations, or acquiring mobile genetic elements like conjugative plasmids, integrons, transposons, or integrating conjugative elements (24).

Our study observed two distinct antibiotic resistance patterns among the cholera isolates of the 2007–2010 and 2015–2016 epidemics. During the 2007–2010 epidemic, 95.5% of strains possessed the SXT elements that harbored the antimicrobial resistance genes *dfrA1*, *sul2*, *strAB*, and *floR*, which are associated with resistance to trimethoprim, sulfamethoxazole, streptomycin, and chloramphenicol, respectively. However, some strains exhibited weak resistance despite carrying multiple resistance genes, which was reported previously and was probably due to lower gene expression levels (25). We found that most of the clinical isolates and two environmental strains in the 2007–2010 epidemic exhibited higher resistance to trimethoprim-sulfamethoxazole (94.6%), streptomycin (92.8%), and nalidixic acid (64.5%). A similar resistance pattern has also been observed in many African and Asian countries (26–31). A recent genomic analysis described that since 2000, most African cholera strains have been carrying SXT elements that harbor antibiotic resistance genes (9). We speculate that the high prevalence of trimethoprim-sulfamethoxazole resistance may be due to its widespread use in preventing opportunistic infections in HIV patients (32). Our data suggest that trimethoprim-sulfamethoxazole, nalidixic acid, and streptomycin are no longer effective drugs for the clinical management of cholera.

Additionally, lower levels of strains resistant to amoxicillin (4.2%), ampicillin (3.6%), chloramphenicol (3%), and doxycycline (1.8%) were observed in the 2007–2010 epidemic, while chloramphenicol intermediate resistance was comparatively high (42.2%). Similar trends were found in the Democratic Republic of Congo and Ghana (33, 34). Scrascia et al. in 2006 reported that V. cholerae O1 strains isolated in Kenya during the 1998–1999 cholera epidemic were resistant to ampicillin, tetracycline, and trimethoprim-sulfamethoxazole (35). Will et al.’s genomic analysis described that nearly all cholera strains in Africa from 1984 to 1998 contained an MDR (incompatibility [Inc]) IncA/C plasmid that may have originated from the outbreak isolates in Tanzania during 1977 that spread to the African Great Lakes region (9). Likewise, tetracycline-resistant strains were observed during Zambia's 1996 to 1997 outbreaks, but it was replaced in 2003 to 2004 (36). Also, in 1979, a cholera outbreak occurred in Bangladesh due to V. cholerae O1 which carried MDR plasmids that conferred resistance to several antibiotics, including tetracycline. This tetracycline disappeared in less than a decade (37, 38). In addition, during the 1988–1989 outbreaks in Bangladesh, nearly all classical V. cholerae strains were resistant to tetracycline, whereas strains belonging to the El Tor biotype were susceptible to the drug (39). This unstable inheritance of El Tor strains due to drug-resistant plasmids has also been reported elsewhere (40). However, in our study, none of the cholera isolates yielded the *tet*(A) gene (tetracycline resistance), probably due to the similar SXT element (ICEVchInd5/ICEVchBan5) backbones that contain no tetracycline resistance genes, and this has remained stable for over a decade in Africa (9, 41). Furthermore, V. cholerae O1 strains isolated from 1994 to 1996 from East African countries, including Kenya, Tanzania, Sudan, Somalia, Rwanda, and Mogadishu, showed an irregular resistance pattern (42). However, the emergence of MDR V. cholerae strains is becoming a significant health problem for regions where cholera is endemic. During the 2007–2010 epidemic, we found that 64.5% of strains were multidrug resistant. This phenomenon has been observed in many countries (24). We found a similar MDR pattern in both the Inaba and Ogawa clinical and environmental strains (Fig. 1; Table S1); this corresponds to the evidence that SXT elements can be transferred between hosts by conjugation and exchange among different serogroups of V. cholerae (16, 30). Our findings suggest that serotype switching has no major effects on the antibiotic resistance pattern (Fig. 1; Table S1). In addition, our study data did not confirm that the source of the cholera epidemic was environmental or due to the importation of the strain by travelers.

Another essential genetic element is integrons, which are responsible for acquiring antibiotic resistance genes in many bacteria, including V. cholerae. These elements are not autonomously mobile but can incorporate gene cassettes, notably those encoding antibiotic resistance, by site-specific recombination (43). Integrons encode an integrase (*intI*) gene that mediates recombination between a sequence in the gene cassette (*attC*) and an attachment site (*attI*) gene. Based on amino acid sequences of *intI*, integrons have been divided into classes, with those carrying *intI*1 defined as class 1 and *intI*2 and *intI*3 as class 2 and 3, respectively. Class 1, 2, and 3 integrons were first identified in association with mobile genetic elements (44). Class 1 integrons are widely distributed among drug-resistant bacteria, including enteric bacteria (14, 45). All the isolates were found negative for class 1, 2, and 3 integrons in our study. Some studies also found the absence of a class 1 integron in cholera strains isolated from outbreaks in Ghana and Iran (34, 45).

The recent epidemic (2015 to 2016) highlighted a low-level resistance pattern observed among cholera strains, as all antibiotics except nalidixic acid were sensitive and no other resistance genes were identified. This was likely due to mobile genetic elements (MGEs) carrying antibiotic resistance gene cassettes not properly dispersed by horizontal gene transfer (15). Iwanaga et al. (15) found that the SXT element of V. cholerae O1 isolated from Laos lost resistance to trimethoprim and gained genes encoding a putative exonuclease and helicase. This demonstrated that SXT elements are unstable and undergo rapid changes.

Furthermore, this study found no evidence of active efflux of quinolone resistance, as no differences in nalidixic acid MIC values were determined by E-test (data not shown). This demonstrated that the mechanism of nalidixic acid resistance in this strain is due to the accumulation of two chromosomal mutations identified in the GyrA (Ser83Ile) and ParC (Ser85Leu). The findings of these two mutations correlate with results of previous studies reported by Kim et al. (46) and Baranwal et al. (47). Treatment with quinolones has recently become a fail-safe choice in the management of cholera and other diarrheal diseases (34).

A limitation of this study is that 2015–2016 cholera epidemic samples were not uniformly collected from the 13 counties. Moreover, inappropriate epidemiological analysis due to incomplete data on gender and age during the 2007–2010 epidemic cannot be ruled out. However, a key target of this study was to identify the antibiotic resistance patterns of cholera outbreak strains, because there was no detailed reported data or literature on drug-resistant patterns and their mechanisms in V. cholerae outbreak isolates in Kenya.

In conclusion, the study evidence suggests that drinking contaminated water contributed to the spread of cholera epidemics in Kenya. Our findings revealed two different antibiotic resistance patterns, indicating that two distinct antibiotic-resistant V. cholerae O1 strains caused the two epidemics. There is a need for continuous monitoring of antibiotic susceptibility testing, which is recommended to determine the emerging antibiotic-resistant V. cholerae strains and their genetic elements for future cholera control strategies.

## MATERIALS AND METHODS

### Bacteria isolation and identification.

A total of 520 stool samples were collected during the 2007–2010 and 2015–2016 cholera epidemics from 13 counties. Two water samples were collected from Pokot County and Kwale County; one water sample was collected from a household water storage container that was collected from a communal shallow well in Pokot County, and another sample was obtained from a water vendor in Kwale County who collected spring water from Shimba Hills and sold it to slum areas. All samples were transported within 24 h of collection and enriched in alkaline peptone water (pH 8.4) at 37°C for 4 to 6 h, followed by overnight culture on thiosulfate citrate bile sucrose agar medium (Eiken, Japan). Sucrose-fermenting yellow colonies were subjected to biochemical assay (48).

### Serogrouping and biotyping.

All V. cholerae strains were confirmed by serotyping with O1 polyvalent and monovalent Inaba/Ogawa antisera (Denka Seiken Co. Ltd., Japan). Biotyping was determined by the Voges Proskauer test, polymyxin sensitivity test, hemolysis of sheep erythrocytes, chicken red blood cell agglutination, and a phase IV test.

### Antimicrobial susceptibility tests.

Antimicrobial susceptibility testing of all isolates against amoxicillin, ampicillin, ceftriaxone, chloramphenicol, ciprofloxacin, doxycycline, gentamicin, nalidixic acid, streptomycin, and trimethoprim-sulfamethoxazole were conducted by determining the MICs using an E-test kit (Liofilchem, Roseto Degli Abruzzi, Italy). The breakpoints recommended by the Clinical and Laboratory Standards Institute guidelines were used for interpretation (49). Escherichia coli ATCC 25922 was used as a control strain. Isolates resistant to three or more classes of antimicrobial agents were defined as multidrug-resistant V. cholerae O1.

### Molecular assay.

PCR was conducted by targeting various virulence and biotype-determining genes for the complement of the phenotypic test (2). The presence of SXT elements, class 1, 2, and 3 integrons, and resistance genes was investigated using the PCR primers listed in Table 3. For screening, the QRDR mutations and the plasmid-mediated quinolone resistance genes *gyrA*, *gyrB*, *parC*, *parE*, and *qnrVC* were amplified and sequenced (46). DNA sequencing was performed using a 3730 DNA analyzer (Applied Biosystems, Foster City, CA, USA). DNA sequences obtained for *gyrA*, *gyrB*, *parC*, and *parE* were compared with the those from a quinolone-susceptible strain of V. cholerae O1 El Tor biotype strain N16961 (GenBank accession number NC_002505).

**TABLE 3:** Primers used in this study

Gene	Sequence (5′–3′)	Reference
SXT (Int)	ATGGCGTTATCAGTTAGCTGGC	50
	GCGAAGATCATGCATAGACC	
*sul2*	AGGGGGCAGATGTGATCGAC	51
	TGTGCGGATGAAGTCAGCTCC	
*dfrA1*	CAAGTTTACATCTGACAATGAGAACGTAT	52
	ACCCTTTTGCCAGATTTGGTA	
*strA*	TTGATGTGGTGTCCCGCAATGC	51
	CCAATCGCAGATAGAAGGCAA	
*strB*	GGCACCCATAAGCGTACGCC	15
	TGCCGAGCACGGCGACTACC	
*floR*	TTATCTCCCTGTCGTTCCAGCG	15
	CCTATGAGCACACGGGGAGC	
*tet*(A)	GTAATTCTGAGCACTGTCGC	53
	CTGCCTGGACAACATTGCTT	
Int1 (class 1 integron)	AAAACCGCCACTGCGCCGTTA	52
	GAAGACGGCTGCACTGAACG	
Int2 (class 2 integron)	ATGTCTAACAGTCCATTTTTAAATTCTA	52
	AAATCTTTAACCCGCAAACGC	
Int3 (class 3 integron)	GTGGCGCAGGGTGTGGAC	52
	ACAGACCGAGAAGGCTTATG	
*gyrA*	AATGTGCTGGGCAACGACTGG	21
	GTGCGCGATTTTCGACATACG	
*gyrB*	GGAAATGACTCGCCGTAAAGG	21
	GTTGTGATAACGCAGTTTATCTGGG	
*parC*	AGAATCTCGGCAAACTTTGACAG	21
	GTCTGAGTTGGGTCTCTCGGC	
*parE*	TTATCGCTGTCAGGGTCAATCC	21
	ATGCGTGCCAGCAAGAAAGTG	
*qnrVC*	AATTTTAAGCGCTCAAACCTCCG	21
	TCCTGTTGCCACGAGCATATTTT	

### Statistical analysis.

Frequencies and percentages were calculated for the study variables. Comparisons were drawn using a two-tailed chi-square test. A *P* value of <0.05 was considered statistically significant.

### Ethics statement.

The study was approved by the KEMRI Scientific and Ethics Review Unit SSC number 1323 and the ethical committee of Nagasaki University Graduate School of Biomedical Sciences.

### Data availability.

The partial sequences of the *gyrA*, *gyrB*, *parC*, and *parE* genes have been deposited in the GenBank database under BioProject numbers OP288101 to OP288104.
